# Pancreatic Mesenchyme Regulates Islet Cellular Composition in a Patched/Hedgehog-Dependent Manner

**DOI:** 10.1038/srep38008

**Published:** 2016-11-28

**Authors:** Daniel Hibsher, Alona Epshtein, Nufar Oren, Limor Landsman

**Affiliations:** 1 Department of Cell and Developmental Biology, Sackler Faculty of Medicine, Tel Aviv University, Tel Aviv 69978 Israel

## Abstract

Pancreas development requires restrained Hedgehog (Hh) signaling activation. While deregulated Hh signaling in the pancreatic mesenchyme has been long suggested to be detrimental for proper organogenesis, this association was not directly shown. Here, we analyzed the contribution of mesenchymal Hh signaling to pancreas development. To increase Hh signaling in the pancreatic mesenchyme of mouse embryos, we deleted *Patched1* (*Ptch1*) in these cells. Our findings indicate that deregulated Hh signaling in mesenchymal cells was sufficient to impair pancreas development, affecting both endocrine and exocrine cells. Notably, transgenic embryos displayed disrupted islet cellular composition and morphology, with a reduced β-cell portion. Our results indicate that the cell-specific growth rates of α- and β-cell populations, found during normal development, require regulated mesenchymal Hh signaling. In addition, we detected hyperplasia of mesenchymal cells upon elevated Hh signaling, accompanied by them acquiring smooth-muscle like phenotype. By specifically manipulating mesenchymal cells, our findings provide direct evidence for the non-autonomous roles of the Hh pathway in pancreatic epithelium development. To conclude, we directly show that regulated mesenchymal Hh signaling is required for pancreas organogenesis and establishment of its proper cellular composition.

The pancreas comprises of exocrine and endocrine cell populations with defined proportions and distinct functions in food digestion and blood glucose regulation, respectively. The majority of pancreatic tissue is populated by exocrine cells, primarily acinar cells, while endocrine cells are organized in islets of Langerhans that are embedded in the exocrine tissue^1^. Islets are composed of α-, β-, δ-, and polypeptide-positive cells, where the predominant cell population is that of β-cells^2^. Pancreas cellular composition is largely dictated during organogenesis, through tightly-regulated cell-specific differentiation and growth rates^3^,^4^,^5^,^6^. These are determined by a combination of intrinsic and extrinsic cues, and are therefore highly dependent on interactions between neighboring cells^3^,^6^,^7^,^8^. Indeed, pancreas organogenesis requires proper interactions of the developing epithelium with its surrounding mesenchyme^7^,^8^,^9^,^10^,^11^,^12^. Although evidence on the importance of the pancreatic mesenchyme in supporting epithelial cell growth and differentiation is accumulating, the question as to whether or not a differential response to mesenchymal cues determines pancreas cellular composition remains open.

During pancreas organogenesis, mesenchymal cells support various developmental stages. The pancreatic epithelium forms from the embryonic foregut endoderm, which recruits adjacent splanchnic mesoderm to form the mesenchymal compartment of the developing organs^3^. At the onset of organogenesis (in mice, around embryonic day (e) 9), the pancreatic bud is populated by common precursors that require mesenchymal cues for their proliferation and survival^7^,^8^,^9^. Later in development (around mouse e12.5–e14.5), as these cells become committed to either an exocrine or endocrine fate^1^,^13^,^14^, their proliferation and maintenance was shown to depend on mesenchymal cues^10^,^11^,^12^. Finally, we previously showed that toward the end of gestation (mouse e16.5–e18.5), the pancreatic mesenchyme supports proliferation of differentiated epithelial cells, including β-cells^11^. However, whether mesenchymal cues differentially affect the distinct epithelial cell types awaits further investigation.

Regulated Hedgehog (Hh) signaling pathway is required for proper pancreas organogenesis^15^,^16^. Activation of this pathway is dependent on the binding of a Hh ligand to its transmembrane receptor, Patched (Ptch)^17^,^18^. The binding of each of the secreted Hh ligands (Sonic (Shh), Indian (Ihh) and Desert (Dhh) hedgehog) releases the repression that Ptch puts on Smoothened (Smo), allowing nuclear localization of the Gli family of transcription factors, and in turn results in expression of Hh-dependent genes^17^. Of note, one of these Hh-dependent genes is *Ptch1*, allowing a negative feedback loop on this pathway^17^. While Hh ligands are highly expressed in adjacent developing organs, namely the stomach and guts, they are absent from the pancreatic epithelium at early stages of development and found in low levels at later stages^19^,^20^,^21^,^22^. Enforced activation of the Hh pathway in the developing pancreas, in mice lacking regulatory elements^23^,^24^ or ectopically expressing Hh ligands in the pancreatic epithelium^19^,^25^, resulted in tissue agenesis, pointing to the essential role of tightly-regulated Hh signaling in pancreas development.

Disrupted Hh signaling affected the development of both endocrine and exocrine cells, and was particularly shown to impair β-cell function and mass^23^,^24^. To differentiate between Hh signaling activation in the pancreas epithelium and its mesenchyme, transgenic mice, in which this pathway was specifically manipulated in the pancreatic epithelium, were generated^22^,^26^,^27^. Regulated Hh signaling within the pancreatic epithelium was shown by others and by us to be required for proper endocrine mass and β-cell function^26^,^27^. Of note, these epithelial-specific manipulations did not fully recapitulate the developmental phenotype observed upon pancreatic-wide manipulation of this pathway^19^,^23^,^24^,^25^. Indeed, it has been long suggested that regulated Hh signaling in the pancreatic mesenchyme is required for proper development^19^. However, the requirement of regulated mesenchymal Hh signaling for pancreas development was not directly shown.

Here, we specifically manipulated pancreatic mesenchymal Hh signaling and studied the resultant effects on epithelial development, focusing on the endocrine pancreas. To this end, we combined the use of *Ptch1*^*flox*^ mice, which allows Cre-dependent deletion of this gene^28^, with *Nkx3.2*-Cre^11^,^29^, expressed by the pancreatic mesenchyme, to generate embryos with elevated levels of Hh signaling in their pancreatic mesenchyme. We observed reduced pancreatic mass in transgenic embryos, when both endocrine and exocrine area were reduced. Interestingly, cellular composition of the endocrine pancreas was disturbed in these embryos when β-cell proportion decreased, indicating it is regulated by Hh-sensitive mesenchymal activity. We further detected hyperplasia of the pancreatic mesenchyme in transgenic embryos, accompanied by abnormal αSMA (α Smooth Muscle Actin) expression. By specifically manipulating mesenchymal cells, our findings provide direct evidence for the non-autonomous roles of the Hh pathway in pancreatic epithelium development.

## Results

### Increased Hh signaling in the embryonic pancreatic mesenchyme upon *Ptch1* deletion

**Figure 1:**
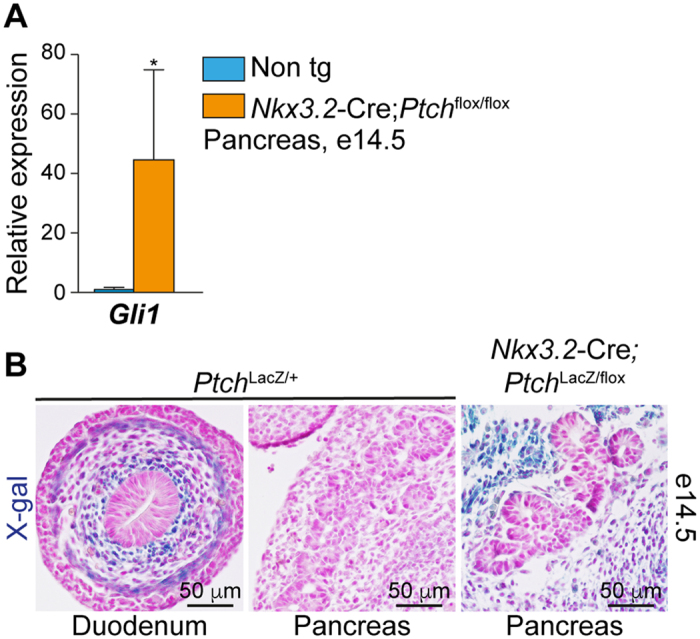
Deletion of *Ptch1* using *Nkx3.2*-Cre mouse line activates Hh signaling in the pancreatic mesenchyme. (**A**) Bar diagram showing *Gli1* expression levels in pancreatic tissues from *Nkx3.2*-Cre;*Ptch*^flox/flox^ (orange bar) and non-transgenic littermate controls (‘non tg’; cyan bar) at embryonic day (e)14.5. Tissues were dissected, their RNA isolated, and gene expression levels were analyzed by qPCR and normalized to non-transgenic controls (n = 3–4). *P < 0.05 (Student’s *t* test). Data represent mean ± SD. (**B**) Dissected gut and pancreatic tissues at embryonic day (e)14.5 incubated with X-gal (blue) and counterstained with Fast Red (pink). Shown are *Ptch*^LacZ/+^ duodenum (left panel) and pancreatic tissue (middle panel), and *Nkx3.2*-Cre;*Ptch*^LacZ/flox^ pancreatic tissue (right panel).

To manipulate Hh signaling in the pancreatic mesenchyme, we set to inhibit the expression of Ptch1, a negative regulator of Hh signaling transduction, which its deletion was shown to increase expression of target genes^17^. To this end, we generated *Nkx3.2*-Cre;*Ptch1*^flox/flox^ embryos, in which the two copies of *Ptch1* are deleted in the *Nkx3.2*-Cre lineage in a Cre/lox dependent manner^28^,^29^. We have previously shown that the *Nkx3.2*-Cre line allows for manipulation of mesenchymal cells, but for no other cell types (including epithelial, endothelial and neuronal) in the developing pancreas^11^. To assess the level of Hh signaling activation, we analyzed the expression levels of *Gli1*, a target gene of this pathway, in the embryonic pancreas of *Nkx3.2*-Cre;*Ptch1*^flox/flox^ and non-transgenic control littermates (*Ptch1*^flox^; Cre-negative) at e14.5. Our analysis revealed increased expression of *Gli1* in pancreatic tissue of transgenic embryos (Fig. 1A), indicating elevated levels of Hh signaling.

To assess the localization of Hh signaling activation, we monitored *Ptch1* expression in the embryonic pancreas. As *Ptch1* is a Hh signaling target gene, *Ptch1*^LacZ^ mouse line, in which a copy of this gene was knocked in by a LacZ cassette, serves as a reporter for Hh signaling activation^30^. In *Ptch1*^LacZ^ e14.5 embryos, LacZ activity is abundant in the mesenchyme surrounding the developing duodenum epithelium (Fig. 1B). However, despite the removal of one copy of *Ptch1*, and in agreement with previous studies^22^,^24^, this activity was below detection in the pancreatic mesenchyme (Fig. 1B). Next, we generated *Nkx3.2*-Cre;*Ptch1*^LacZ/flox^ compound embryos, in which one copy of *Ptch1* was knocked in by the LacZ transgene, and the other (*Ptch1*^flox^) depleted in the *Nkx3.2*-Cre lineage, as described above. Analysis of LacZ activity in *Nkx3.2*-Cre;*Ptch1*^LacZ/flox^ e14.5 embryos pointed to increased Hh signaling in their pancreatic mesenchyme, but not in the epithelium (Fig. 1B). Thus, deleting *Ptch1* using the *Nkx3.2*-Cre mouse line allowed increased mesenchymal Hh signaling in the developing pancreas.

### Deletion of *Ptch1* in the pancreatic mesenchyme results in reduced pancreatic mass

**Figure 2:**
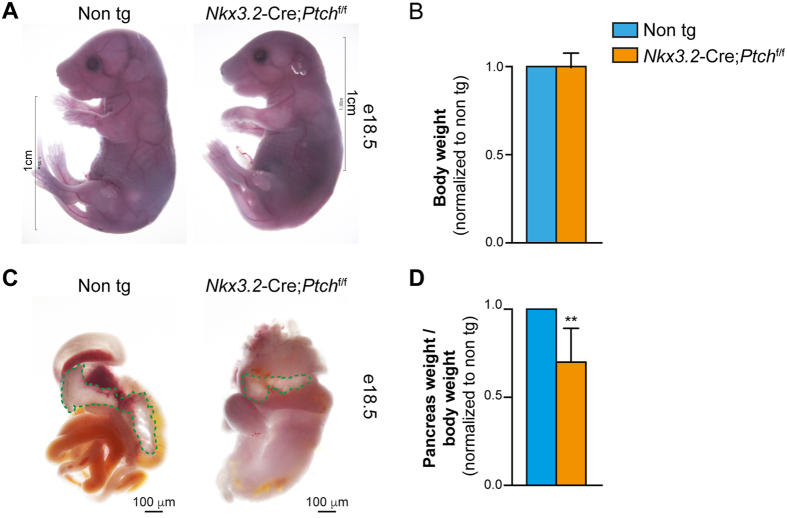
Reduced pancreatic mass upon increased mesenchymal Hh signaling. *Nkx3.2*-Cre;*Ptch*^flox/flox^ transgenic and non-transgenic (Cre negative; ‘Non tg’) littermate embryos were analyzed at e18.5. (**A**) Whole body images of transgenic embryo (right) and non-transgenic littermate (left). (**B**) Bar diagram (mean ± SD) summarizing normalized body weight of transgenic (orange bar) to non-transgenic (cyan bar; set to ‘1’) littermates. n = 5. (**C**) Images show gross morphology of dissected embryonic gastrointestinal tract from transgenic (right) and non-transgenic (left) littermates. Pancreatic tissue is outlined with a green dashed line. (**D**) Bar diagram (mean ± SD) summarizing normalized pancreas weight of transgenic (orange bar) to non-transgenic (cyan bar; set to ‘1’) littermates. n = 5. p value: **P < 0.01, as compared to non-transgenic control, determined using Student’s *t*-test.

To analyze the resultant effect of increased mesenchymal Hh signaling on pancreatic development, we analyzed *Nkx3.2*-Cre;*Ptch1*^flox/flox^ embryos and non-transgenic control littermates (*Ptch1*^flox^; Cre-negative). *Nkx3.2*-Cre;*Ptch1*^flox/flox^ mice died upon birth, likely due to non-pancreatic expression of the *Nkx3.2*-Cre line in the embryonic gut and stomach mesenchyme and in skeletal somites^29^,^31^. However, at e18.5 transgenic embryos exhibited comparable appearance and body weight to their non-transgenic control littermates (Fig. 2A and B). As previously reported upon elevated Hh signaling in the *Nkx3.2*-Cre lineage^32^, the gastrointestinal tract of transgenic mice was drastically deformed, with short and dilated intestine and misshapen stomach (Fig. 2C). Of note, we were unable to detect splenic tissue, a derivative of the embryonic pancreatic mesenchyme^33^, in transgenic mice. Nonetheless, pancreatic tissue could be detected in *Nkx3.2*-Cre;*Ptch1*^flox/flox^ embryos (Fig. 2C). Pancreatic tissue of transgenic embryos was significantly smaller than that of their non-transgenic littermates (Fig. 2D), implicating that increased Hh signaling in the pancreas mesenchyme affects proper organogenesis.

### Elevated Hh signaling leads to expansion of the pancreatic mesenchyme

**Figure 3:**
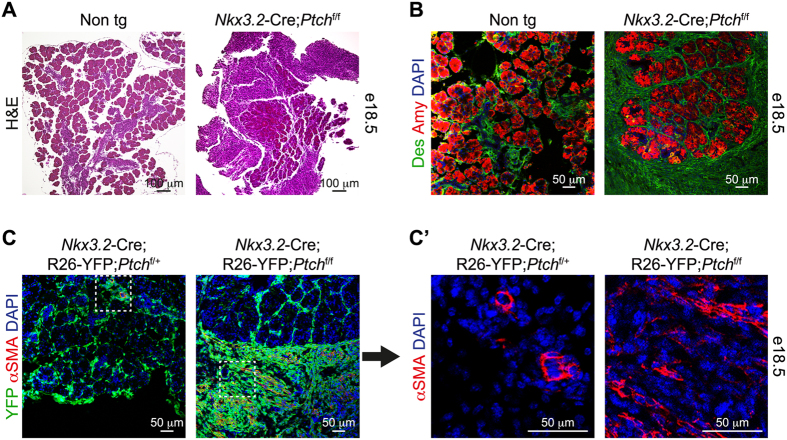
Hyperplasia of the pancreatic mesenchyme upon elevated Hh signaling. (**A**) Histological analysis of pancreatic tissue of e18.5 *Nkx3.2*-Cre;*Ptch*^flox/flox^ transgenic (right) and non-transgenic (Cre negative; ‘Non tg’; left) littermate embryos. Tissue-sections stained with Hematoxylin and Eosin (H&E). (**B**) Pancreatic tissues of e18.5 *Nkx3.2*-Cre;*Ptch*^flox/flox^ transgenic and non-transgenic (Cre negative; ‘Non tg’) littermate embryos were stained with antibodies against Desmin (‘Des’; green) and Amylase (‘Amy’; red) and counterstained with DAPI (blue). Shown are representative fields. (**C**) Immunofluorescence analysis of dissected pancreatic tissues from *Nkx3.2*-Cre;R26-YFP;*Ptch*^flox/flox^ (right) and *Nkx3.2*-Cre;R26-YFP;*Ptch*^flox/+^ (left) control littermate e18.5 embryos. Tissues were stained with antibodies against YFP (green), α smooth muscle actin (αSMA; red) and counterstained with DAPI (blue). C’) Higher magnification of areas framed in a white box in (**C**) showing the αSMA and DAPI channels. Shown are representative fields.

To further analyze the effect of deregulated mesenchymal Hh signaling on pancreas development, we dissected pancreatic tissue from e18.5 *Nkx3.2*-Cre;*Ptch1*^flox/flox^ and non-transgenic littermate control embryos. While non-transgenic pancreatic tissue contained mostly epithelial cells, histological analysis revealed abundant non-epithelial cells in pancreatic tissue of *Nkx3.2*-Cre;*Ptch1*^flox/flox^ embryos (Fig. 3A).

Hh signaling was shown to support proliferation of mesenchymal cells lining the developing gastrointestinal tract^32^. In order to test if elevated Hh signaling leads to changes in the mesenchymal cell layer, we labeled these cells using the pan-mesenchymal marker desmin. In control embryos, desmin-expressing cells formed a thin, cell-wide layer surrounding acinar lobes (Fig. 3B). In contrast, transgenic pancreatic tissues displayed multi-cellular desmin-positive mesenchymal layer that extended away from the epithelium (Fig. 3B). Of note, expanded mesenchymal layer was detected also in between acinar cells. Thus, our results point to hyperplasia of the pancreatic mesenchyme upon increased Hh signaling in these cells.

Pancreatic mesenchymal cells were shown to acquire smooth muscle fate, typical to mesenchymal cells surrounding the gut, in response to increased Hh signaling^19^. To study potential changes in these cells, we employed the R26R-YFP transgenic mouse line, which allows for YFP expression in a Cre-dependent manner^34^. As *Nkx3.2*-Cre express mesenchymal cells from early stages of pancreas development^11^,^31^, the inclusion of a Cre-dependent YFP reporter allowed us to trace these cells, regardless of potential phenotypical changes. To analyze for potential acquisition of smooth muscle fate upon elevated mesenchymal Hh signaling, we analyzed the expression of αSMA in YFP-expressing cells. As expected, in control *Nkx3.2*-Cre;*R26R*-EYFP;*Ptch1*^flox/+^ pancreatic tissue, αSMA expression was observed in vascular smooth muscle cells (vSMCs), embedded in the pancreatic tissue (Fig. 3C and C’)^35^. However, in *Nkx3.2*-Cre;*R26R*-EYFP;*Ptch1*^flox/flox^ embryos, this marker was expressed by YFP-expressing cells that extended away from the pancreatic epithelium in transgenic embryos (Fig. 3C and C’). Furthermore, the fusiform shape of αSMA-expressing cells (Fig. 3C’) further suggests pancreatic mesenchymal cells acquire smooth muscle-like phenotype upon deregulated Hh signaling, as previously suggested^19^.

To conclude, in agreement with previous reports^19^,^32^, we observed hyperplasia of the pancreatic mesenchyme upon elevated Hh signaling, accompanied by acquisition of smooth muscle-like phenotype.

### Reduced epithelial area upon deletion of *Ptch1* in the pancreatic mesenchyme

**Figure 4:**
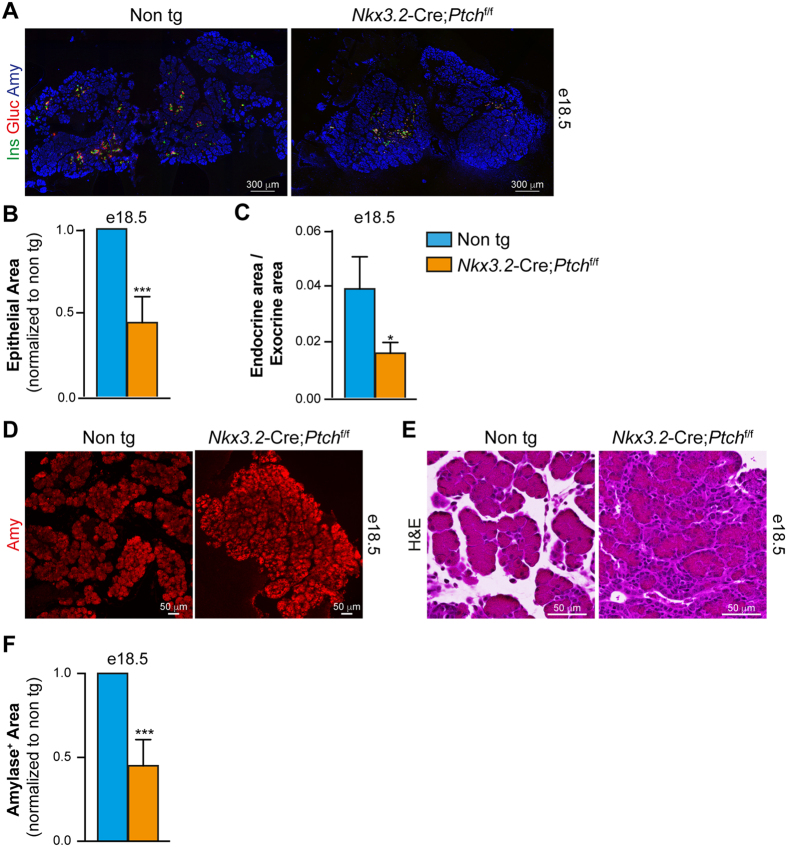
Reduced pancreatic epithelium upon increased mesenchymal Hh signaling. (**A**) Immunofluorescence analysis of dissected pancreatic tissues from *Nkx3.2*-Cre;*Ptch*^flox/flox^ (right panel) and non-transgenic (left panel) stained with antibodies against insulin (‘Ins’; green), glucagon (‘Gluc’; red) and amylase (‘Amy’; blue). (**B**) Bar diagram (mean ± SD) shows epithelial area (combined glucagon, insulin, and amylase-positive areas) in transgenic (orange bar) and non-transgenic (cyan bar) littermates. n = 3. p value: ***p < 0.005, as compared to non-transgenic control, determined using Student’s *t*-test. (**C**) Bar diagram (mean ± SD) shows endocrine area (combined glucagon and insulin -positive areas) divided by exocrine area (amylase-positive area), in transgenic (orange bar) and non-transgenic (cyan bar) littermates. n = 3. p value: *p < 0.05, as compared to non-transgenic control, determined using Student’s *t*-test. (**D**) Immunofluorescence analysis of dissected pancreatic tissues from *Nkx3.2*-Cre;*Ptch*^flox/flox^ (right panel) and non-transgenic (non tg; left panel) stained with an antibody against amylase (‘Amy’; red). Shown are representative fields. (**E**) Histological analysis of pancreatic tissue of e18.5 *Nkx3.2*-Cre;*Ptch*^flox/flox^ transgenic (right panel) and non-transgenic (‘Non tg’; left panel) littermate embryos. Tissue-sections stained with Hematoxylin and Eosin (H&E). Shown are representative fields. (**F**) Bar diagram (mean ± SD) shows Amylase-positive area in transgenic (orange bar) and non-transgenic (cyan bar) littermates. n = 3. p value: ***p < 0.005, as compared to non-transgenic control, determined using Student’s *t*-test.

To analyze the resultant effect of increased mesenchymal Hh signaling on pancreatic epithelial development, we analyzed for the presence of the most abundant pancreatic epithelial cell types, β-, α- and acinar cells, in *Nkx3.2*-Cre; *Ptch1*^flox/flox^ and non-transgenic littermate controls at e18.5. Immunofluorescence analysis revealed the presence of all three cell types in transgenic embryos (Fig. 4A), with endocrine cells embedded in exocrine tissue. However, morphometric analysis revealed that the combined area of the three epithelial cell population was significantly smaller in transgenic embryos (Fig. 4B). Note that the reduction of epithelial area was more profound than the reduction in pancreatic weight, likely representing the contribution of mesenchyme hyperplasia to the latter (compared Figs 2D and 4B). In addition, the ratio between endocrine (combined insulin- and glucagon-positive area) and exocrine area (amylase-positive area) was smaller in transgenic embryos (Fig. 4C), implicating these two cellular compartments were differentially affected by increased mesenchymal Hh signaling.

Morphology of exocrine tissue was disrupted in transgenic embryos, with more compacted cellular distribution (Fig. 4A,D and E). Our analysis indicated that the typical acellular areas normally found between adjacent lobes is lost in transgenic embryos, and is filled by mesenchymal cells (Figs 3B and 4E). As expected from the reduced epithelial area, morphometric analysis revealed smaller amylase-positive area in *Nkx3.2*-Cre;*Ptch1*^flox/flox^ e18.5 embryos as compared to littermate control (Fig. 4F). To conclude, our findings indicate that deregulated mesenchymal Hh signaling impairs growth and morphology of the exocrine pancreas.

### Abnormal islet morphology in *Nkx3.2*-Cre;*Ptch1* ^flox/flox^ pancreas

**Figure 5:**
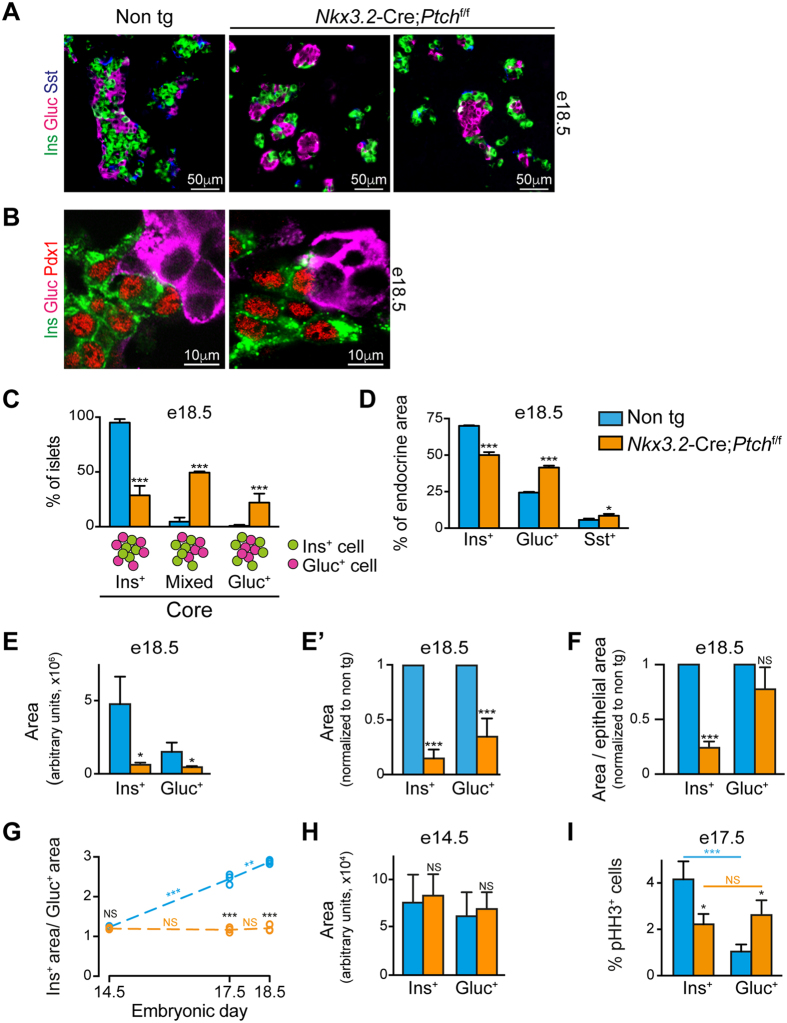
Abnormal endocrine development upon increased mesenchymal Hh signaling. Pancreatic tissues from *Nkx3.2*-Cre;*Ptch*^flox/flox^ transgenic (orange) and non-transgenic (‘Non tg’; cyan) littermate embryos were analyzed at indicated embryonic days. (**A**,**B**) Immunofluorescence analysis of dissected e18.5 pancreatic tissues stained with antibodies against insulin (‘Ins’; green), glucagon (‘Gluc’; magenta) and either somatostatin (‘Sst’; blue) (**A**) or Pdx1 (red)(**B**). Shown are representative fields. (**C**) Bar diagram (mean ± SD) shows the percentage of islets with insulin-positive (‘Ins^+^ core’; left bars), glucagon-positive (‘Gluc^+^ core’; right bars) core, or core made of both cell types (‘Mixed’; middle bars) at e18.5. Cartoon show illustration of islets with indicated cores. n = 3. (**D**) Bar diagrams (mean ± SD) show the percentage of insulin (Ins^+^), glucagon (Gluc^+^) and somatostatin (Sst^+^) positive areas of total endocrine area (set as the combined area of all three cell types) at e18.5. n = 3. (**E,E’**) Bar diagram (mean ± SD) shows measurement of insulin (Ins^+^) and glucagon (Gluc^+^) positive areas at e18.5, represented in arbitrary units (**E**) or normalized to non-transgenic littermate control (non tg; set to 1)(E’). n = 3. (**F**) Bar diagram (mean ± SD) shows measurement of insulin (Ins^+^) and glucagon (Gluc^+^) positive areas at e18.5, divided by total epithelial area (as shown in Fig. 4B) and normalized to non-transgenic control (non tg; set to 1). n = 3. (**G**) Scattered dot plot showing the ratio between insulin (Ins^+^) and glucagon (Gluc^+^) positive areas in pancreatic tissues of e14.5, e17.5 and e18.5 embryos. Dashed lines represent trend lines between adjacent ages. (**H**) Bar diagram (mean ± SD) shows measurement of insulin (Ins^+^) and glucagon (Gluc^+^) positive areas at e14.5, represented in arbitrary units. n = 3. (**I**) Bar diagram (mean ± SD) shows the percentage of insulin (Ins^+^), glucagon (Gluc^+^) –positive cells co-expressing phosphorylated Histone H3 (pHH3). n = 3. P values: *p < 0.05, **p < 0.01, ***p < 0.005, NS = non-significant. Comparison between transgenic and non-transgenic embryos are marked with black font, while comparison between samples of the same genotype are marked with orange (transgenic embryos) and cyan (non-transgenic embryos) fonts, all determined using student’s *t*-test.

Our analysis indicates that endocrine mass is affected more so than that of exocrine from elevated Hh signaling (Fig. 4C). To analyze for potential changes in endocrine cells upon mesenchymal *Ptch1* deletion, pancreatic tissues of *Nkx3.2*-Cre;*Ptch1*^flox/flox^ and non-transgenic littermates e18.5 embryos were immune-stained for insulin, glucagon, and somatostatin. Our analysis revealed that while all three endocrine cell populations are present in transgenic embryos, islet morphology was abnormal (Fig. 5A). Of note, Pdx1 expression by insulin-positive, but not by glucagon-positive cells in transgenic pancreatic tissue pointed to appropriate cell fate acquisition^36^ (Fig. 5B).

In mice, β-cells populate the islet core, while α- and δ-cells are found in the islet periphery, forming a mantle^2^. As shown in Fig. 5A, transgenic islets displayed a disrupted organization, with some having a α-cell core and β- and δ-cell mantle. To quantify the observed morphological changes, we divided islets into three groups based on their core: distinct β-cell core, distinct α-cell core, or with a core formed by both cell types (‘mixed’; Fig. 5C). A vast majority of islets in non-transgenic pancreatic tissue had the typical β-cell core, with few having a core containing both β- and α-cells (Fig. 5C). In contrast, less than one-third of islets in e18.5 *Nkx3.2*-Cre;*Ptch1*^flox/flox^ pancreatic tissue had a defined β-cell core, with about one-half of their islets exhibited a core containing both β- and α-cells. Interestingly, while we observed no islets with a α-cell core in non-transgenic pancreatic tissues, around one-fifth of transgenic islets had a distinct α-cell core (Fig. 5C). Thus, our findings indicate that deregulated mesenchymal Hh signaling leads to disrupted islet morphology.

### Abnormal endocrine composition and mass upon deletion of mesenchymal *Ptch1*

To analyze if the observed abnormal islet morphology is associated with changes in islet cellular composition, we directly compare the portion of endocrine cell types. To this end, we measured insulin^+^, glucagon^+^ and somatostatin^+^ area in pancreatic tissues of *Nkx3.2*-Cre;*Ptch1*^flox/flox^ transgenic and control e18.5 embryos. As shown in Fig. 5D, non-transgenic tissues exhibit the expected endocrine cell ratio^2^, with ~70% being insulin^+^, ~24% being glucagon^+^ and the remaining ~6% being somatostatin^+^. However, transgenic pancreatic tissues had a reduced insulin^+^ portion to ~50% of endocrine cells, whereas the portion of glucagon^+^ increased to ~41% and that of somatostatin^+^ to ~9% of endocrine cells (Fig. 5D). To conclude, our findings indicate that regulated mesenchymal Hh signaling is required to maintain the stereotypical islet cellular composition.

To directly analyze for potential changes in α- and β-cell mass upon deregulation of mesenchymal Hh signaling, we analyzed their area. Morphometric analysis revealed a significantly reduced area in both these cell populations in e18.5 *Nkx3.2*-Cre;*Ptch1*^flox/flox^ embryos, as compared to littermate control (Fig. 5E and E’). Furthermore, while the reduction of glucagon^+^ area was proportional to the reduced epithelial area in transgenic embryos, the reduction in insulin^+^ area was more profound than the reduction in total epithelial area (Fig. 5F). In agreement with other studies^23^,^24^,^25^, our analysis indicates that β-cell mass is affected more so than that of α-cells from elevated Hh signaling.

### Regulated mesenchymal Hh signaling is required for endocrine cell-specific proliferation rates

Our analysis implicates that manipulating Hh signaling in the pancreatic mesenchyme differentially affected α- and β- cell development. To directly test this possibility, we calculated the ratio between insulin^+^ and glucagon^+^ area at e14.5, e17.5, and e18.5 in *Nkx3.2*-Cre; *Ptch1*^flox/flox^ embryos and littermate controls. As shown in Fig. 5G, in control embryos, insulin^+^ area was slightly larger (by ~1.2 fold) than glucagon^+^ area at e14.5, likely representing increased frequency of precursor differentiation toward a β-cell fate^13^, combined with larger β-cell size (Table 1)^37^. However, insulin/glucagon ratio significantly increased with age in non-transgenic embryos; while insulin^+^ area was 2.4-fold bigger than glucagon^+^ area at e17.5, this difference grew to 2.9-fold at e18.5 (Fig. 5G). This analysis indicates that β-cell population grows at a higher rate than α-cell population during normal pancreas development.

In e14.5 *Nkx3.2*-Cre;*Ptch1*^flox/flox^ embryos, insulin/glucagon ratio was comparable to that of control (ratio of 1.2) (Fig. 5G). Of note, transgenic embryos displayed comparable insulin^+^ and glucagon^+^ area to their littermate controls at this age (Fig. 5H). Interestingly, this ratio remained constant in transgenic embryos (ratio of ~1.2) at the three analyzed ages (Fig. 5G), implicating similar growth rates of the two cell populations upon deregulated mesenchymal Hh signaling.

Our analysis indicates that β-cell population grows at a higher rate than the α-cell population during normal development, but not upon increased mesenchymal Hh signaling (Fig. 5G). As this is observed also between e17.5 and e18.5, after differentiation of the two populations from common endocrine precursors had ceased^1^, we analyzed for potential differences in cell proliferation at e17.5. To this end, we analyzed the portion of α- and β- cells expressing the proliferation marker phosphorylated Histone H3 (pHH3) in *Nkx3.2*-Cre;*Ptch1*^flox/flox^ and non-transgenic e17.5 embryos. As shown in Fig. 5I, the portion of pHH3^+^ β-cells in non-transgenic embryos was significantly higher (~4-fold) than that of α-cells. In contrast, the portions of pHH3^+^ β-cells and α-cells in transgenic embryos were comparable (Fig. 5I). Of note, while the rate of β-cell proliferation at e17.5 decreased in transgenic embryos as compared to control, the rate of α-cell proliferation increased.

This set of experiments indicate that during normal pancreas development, β-cells grow at a higher rate than α-cells to establish proper islet cellular composition. Furthermore, our results suggest that this cell specific growth rates require regulated Hh signaling in mesenchymal cells.

### Deregulated Hh signaling leads to elevated mesenchymal mass

**Figure 6:**
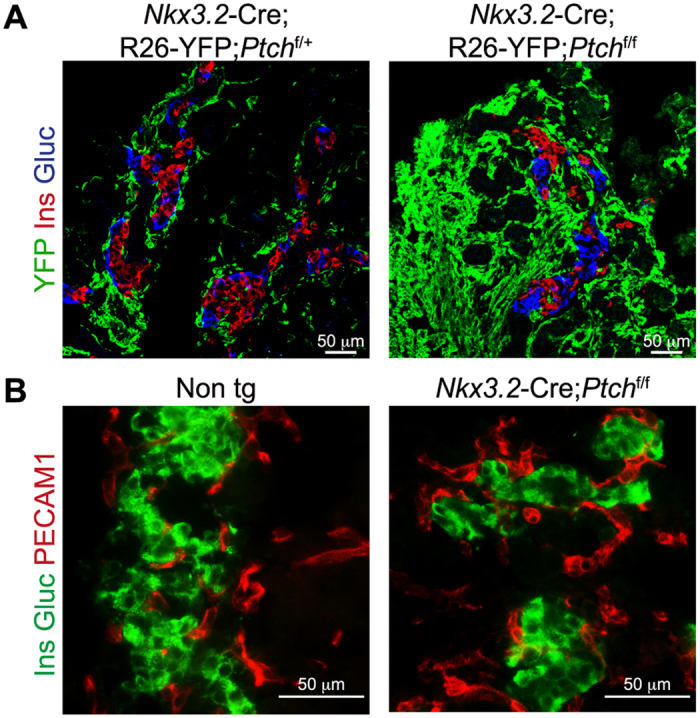
Deregulated Hh signaling leads to mesenchymal hyperplasia. (**A**) Immunofluorescence analysis of dissected pancreatic tissues from *Nkx3.2*-Cre;R26-YFP;*Ptch*^flox/flox^ (right) and *Nkx3.2*-Cre;R26-YFP;*Ptch*^flox/+^ (left) control littermate e18.5 embryos. Tissues were stained with antibodies against YFP (green), insulin (‘Ins’; red) and glucagon (‘Gluc’; blue). Shown are representative fields. (**B**) Immunofluorescence analysis of dissected pancreatic tissues from *Nkx3.2*-Cre; *Ptch*^flox/flox^ transgenic (right) and non-transgenic (Non tg; left) stained with antibodies against insulin (‘Ins’; green), glucagon (‘Gluc’; green) and the endothelial marker PECAM1 (red). Shown are representative fields.

Mesenchymal cells surround islet of Langerhans, and their depletion abrogated endocrine cell growth^11^. To analyze whether endocrine-associated mesenchymal cells are affected by increased Hh signaling, pancreatic tissues of *Nkx3.2*-Cre;*R26R*-EYFP;*Ptch1*^flox/flox^ and *Nkx3.2*-Cre;*R26R*-EYFP;*Ptch1*^flox/+^ littermate control e18.5 embryos were immuno-stained for YFP, insulin, and glucagon. As shown in Fig. 6A, while mesenchymal cells formed a thin, cell-wide layer surrounding the islet of Langerhans in control embryos^11^, this layer was considerably thickened in *Nkx3.2*-Cre;*R26R*-EYFP;*Ptch1*^flox/flox^ embryos (Fig. 6A), similarly to the observed thickening of this layer in the exocrine tissue (Fig. 3).

Endothelial cells were shown to regulate β-cell development^38^,^39^. Although endothelial cells are not targeted by the *Nkx3.2*-Cre mouse line^11^,^40^, they might be affected by changes in the pancreatic mesenchyme. We therefore stained pancreatic tissues of *Nkx3.2*-Cre;*Ptch1*^flox/flox^ and non-transgenic e18.5 embryos for the endothelial marker Platelet Endothelial Cell Adhesion Molecule 1 (PECAM1). Our analysis indicated the presence of endothelial cells in and around islets of both transgenic and control embryos, with similar distribution (Fig. 6B). To conclude, our findings point to increased mesenchymal mass, without apparent change in endothelial area, around islet of Langerhans upon deregulated Hh signaling.

## Discussion

Regulation of Hh signaling is essential for proper pancreas development. Here, we analyzed the contribution of mesenchymal Hh signaling to this process. Our results indicate that the ability of the pancreatic mesenchyme to support organogenesis depends on proper regulation of Hh signaling in these cells. To increase Hh signaling in the pancreatic mesenchyme, we generated *Nkx3.2*-Cre;*Ptch1*^flox/flox^ embryos, in which two copies of *Ptch1* were deleted in this tissue. Deregulated Hh signaling in mesenchymal cells was sufficient to disrupt epithelial growth, affecting both the endocrine and the exocrine pancreas. However, mesenchymal growth was increased, leading to hyperplasia of this cell layer. We further observed disrupted endocrine cellular composition, with a reduced β-cell portion and abnormal islet morphology. Thus, our findings indicate that the cell-specific growth rates of epithelial cell populations depend on the pancreatic mesenchyme, and requires regulated Hh signaling activity in this cell layer. To conclude, we showed that mesenchymal Hh signaling is required for pancreatic growth and establishment of its cellular composition.

Islets of Langerhans display a characteristic cellular composition, determined during development^2^,^13^. Our results indicate that in the mouse embryo, pancreatic endocrine cells exhibit specific growth rates, with the β-cell population growing at a higher rate than the α-cell population. In part, this could be an outcome of a higher tendency of endocrine precursors to differentiate to β-cells than to alternative cell fates^13^. In addition, our results suggest that cell-specific proliferation rate might contribute to the stereotypical islet composition, when β-cells proliferate at a higher rate than α-cells do. Deregulated Hh signaling in pancreatic mesenchymal cells, achieved by deletion of *Ptch1* in these cells, led to similar β- and α- cell growth rates toward end of gestation, likely contributing to the observed abnormal islet composition. While we observed abnormal cell proliferation rates in transgenic embryos, this could not fully explain the dramatic reduction in β- and α-cell mass. It is therefore possible that endocrine cells proliferate at a higher rate at earlier developmental stages. Alternatively, although normal β- and α- mass was observed at e14.5, their differentiation rate was affected by deregulated mesenchymal Hh signaling. Of note, β-cell development was shown by others to be more affected than α-cells from deregulated pancreatic Hh^25^, further suggesting their specific growth rate is dependent on restrained Hh signaling. While β-cell function was shown to require cells in the islet microenvironment^38^,^40^, the postnatal lethality of *Nkx3.2*-Cre;*Ptch1*^flox/flox^ mice prevents us from being able to directly study the role of mesenchymal Hh signaling in this process. Nevertheless, the abnormal endocrine composition observed in transgenic mice would have likely affected the levels of secreted hormones.

Mouse islets have a distinct β-cell core, whereas the core of human islets were reported to be populated by both α- and β-cells^2^,^41^. Recent studies suggest that the morphology of human islets depends on their size, whereas small islets resemble the morphology of mouse islets, with a distinct β-cell core^42^,^43^. Furthermore, the arrangement of endocrine cells within the islets allows for proper homotypic and heterotypic interactions, essential for proper functioning^2^,^44^. We observed that increased mesenchymal Hh signaling leads to abnormal islet organization, with most islets lacking the typical β-cell core. It could therefore be possible that the α- to β- cell ratio dictates islet morphology, and that the abnormal endocrine cell ratio in transgenic embryos leads to the abnormal islet morphology. This possibility is supported by the abnormal islet morphology observed upon increased β-cell death during development^45^. Alternatively, similarly to neurons^46^, mesenchymal cells may directly dictate pancreatic endocrine cell arrangement and islet morphology.

Pancreas morphogenesis and growth depend on proper interactions of the developing epithelium with cells in its surrounding. Mesenchymal-epithelial interaction was shown to be promote epithelial branching^11^,^47^. Our findings indicate that deregulated mesenchymal Hh signaling leads to hyperplasia of mesenchymal cells and abrogated epithelial expansion and branching. The pancreatic endothelium was shown to restrain exocrine growth, when hyper-vascularization repress pancreas expansion and branching^48^,^49^,^50^. It is therefore possible that, similarly to endothelial cells, mesenchymal hyperplasia does not allow proper mesenchymal-epithelial interactions, leading to the observed morphological phenotype. Alternatively, yet to be identified Hh-dependent mesenchymal cues may regulate pancreatic growth and branching.

The role of Hh signaling in pancreas development was established by a series of studies that manipulated this pathway in both epithelial and mesenchymal compartments^19^,^21^,^23^,^24^,^25^. These studies showed that restrained pancreatic Hh signaling is crucial for epithelial expansion and proper β-cell mass and function. Epithelial and β-cell specific manipulations of this pathway recapitulated some of these phenotypes, including reduced endocrine mass and impaired β-cell function, indicating a cell intrinsic role of Hh signaling^22^,^26^,^27^. By manipulating this pathway in the pancreatic mesenchyme, but not in its epithelium, we directly showed a cell extrinsic roles of Hh signaling in epithelial expansion. Of note, we could not observe ectopic pancreas in *Nkx3.2*-Cre;*Ptch1*^f/f^ embryos, further indicating that this phenomena is an outcome of deregulated Hh signaling in the pancreatic epithelium^51^. Importantly, neither epithelial nor mesenchymal -specific elevation of Hh signaling fully recapitulate the pancreatic agenesis phenotype observed upon pancreas-wide manipulation of this pathway^19^,^23^,^24^,^25^. The different manipulations of this pathway (removal of regulatory elements, ectopic ligand, and transcription factors’ expression) may lead to different levels of pathway activation. Alternatively, deregulating Hh signaling in both the epithelium and the mesenchyme might have a synergetic, negative effect on epithelial growth. The severity of pancreatic phenotype observed upon systemic manipulation of *Ptch1* expression^24^, as compared to the phenotype described here upon mesenchymal manipulation of this gene, supports the requirement of regulated Hh signaling in both pancreatic epithelium and mesenchyme.

Hh signaling was shown to be required for proliferation of mesenchymal cells of the gastrointestinal tract^32^. While along the gut tube mesenchymal cells form the smooth muscle layer that controls its local movement, the adult pancreas lacks this layer and contains relatively few mesenchymal cells (including pancreatic stellate cells, vSMCs, and pericytes)^35^,^52^. Therefore, the expression of Hh ligands along the gut tube, and their exclusion from the developing pancreas, may reflect a differential need for mesenchymal expansion^19^,^20^,^32^. This notion was first suggested by Apelqvist and colleagues in 1997, in a seminal study reporting acquisition of a gut-like phenotype by pancreatic mesenchymal cells upon ectopic Shh expression^19^, and was further supported by others^23^,^24^,^25^. Furthermore, Hh signaling was shown to promote stroma expansion during the progression of pancreatic ductal adenocarcinoma (PDAC)^53^. Here, we were able to directly show that elevated Hh signaling leads to expansion of the mesenchymal layer in a cell-autonomous manner. Hence, regulated Hh signaling may be required for establishing a proper epithelial-mesenchymal ratio in the digestive system, allowing for proper size and functioning of these organs.

## Materials and Methods

### Mice

All experiments were performed according to protocols approved by the Committee on Animal Research at Tel Aviv University. *Nkx3*.2 (*Bapx1*)-Cre (*Nkx3–2*^tm1(cre)Wez^)^29^, *Ptch1*^LacZ^ (*Ptch1*^tm1Mps/J^)^30^, *Ptch1*^flox^ (*Ptch1*^tm1Bjw^)^28^, *R26*-YFP (Gt(ROSA)26Sor^tm1(EYFP)Cos/J^)^34^ mouse lines were used in this study. Noon on the day a vaginal plug was detected was considered as embryonic day 0.5.

### X-gal staining

Dissected gastrointestinal tissues were fixed with Paraformaldehyde (4%) for 2 h and incubated overnight at room temperature with X-gal (5-Bromo-4-chloro-3-indolyl β-D-galactopyranoside; 2.5 mg/ml; Sigma) diluted in PBS containing Potassium Ferrocyanide (4.35 mM), Potassium Ferricyanide (5 mM), NP-40 (0.02%), MgCl_2_ (2 mM), followed by a second round of fixation in Paraformaldehyde (4%) overnight. Tissues were then embedded in paraffin, sectioned, and counter-stained with nuclear Fast Red (Vector). Images were acquired using Keyence BZ-9000 microscope (Biorevo).

### Hematoxylin and Eosin staining

Dissected pancreatic tissues were fixed with Paraformaldehyde (4%) for 2 hours, embedded in paraffin wax and sectioned. Following deparaffinization, tissue sections were stained with Meyer’s Hematoxylin (Sigma) followed by staining with Eosin (Sigma). Images were acquired using Keyence BZ-9000 microscope (Biorevo).

### Immunofluorescence

Dissected pancreatic tissues were fixed with Paraformaldehyde (4%) for 2–4 hours. Tissue were embedded in paraffin wax, sectioned to 5 μm sections and stained using the following primary antibodies: rabbit anti-Amylase (Sigma, Catalog #A8273), rabbit anti-Glucagon (Millipore, Catalog #AB932), mouse anti-Glucagon (Sigma, Catalog #G2654), guinea pig anti-Insulin (DAKO, Catalog #A0564), rabbit anti-phosphorylated Histone H3 (Millipore, Catalog #06–570), rat anti-Somatostatin (Millipore, Catalog #MAB354). Alternatively, following their fixation, tissues were embedded in Tissue-Tek O.C.T. Compound (Sakura Finetek), cryo-sectioned to 11 μm sections and stained using the following primary antibodies: mouse anti-Desmin (Dako; Catalog #M0760), rabbit anti-Glucagon (Millipore, Catalog #AB932), mouse anti-Glucagon (Sigma, Catalog #G2654), guinea pig anti-Insulin (DAKO, A0564), rabbit anti-Pdx1 (Millipore, Catalog #MM07696), rat anti-PECAM1 (BD, Catalog #553370), anti-αSMA (Abcam, Catalog #Ab5694) and chicken anti-YFP/GFP (Abcam, Catalog #Ab13970). Staining was followed by staining with AlexaFluor tagged secondary antibodies (1:500, Invitrogen) and mounting with DAPI-containing Vectashield media (Vector). Images were acquired using Keyence BZ-9000 microscope (Biorevo) and SP8 confocal microscope (Leica).

### Morphometric quantifications

For all measurements presented in this study the following regimen was applied: the entire pancreatic tissue, including both dorsal and ventral buds, was embedded in paraffin wax and cut into 5 μm thick sections. For e18.5 and e17.5 embryos, every fifth section (20% of total tissue) was immuno-stained with indicated antibodies (as described above), where each transgenic tissue was processed and stained in parallel with its littermate control. For e14.5 embryos, half of the sections was immuno-stained with indicated antibodies (as described above), where each transgenic tissue was processed and stained in parallel with its littermate control. Sections were automatically imaged using Keyence BZ-9000 microscope (Biorevo). For all quantifications, with the exception of the measurement of islet morphology and pHH3 expression, all acquired images were analyzed using imageJ software (NIH). For analysis of islet morphology, 50–70 islets from each embryo were manually scored, blind to genotype. For analysis of percentage of pHH3 expressing cells, at least 150 cells from each analyzed cell type in each embryo were manually scored, blind to genotype.

### Quantitative PCR

RNA was extracted from isolated tissues using PureLink RNA Micro Kit (Invitrogen), followed by a reverse transcription reaction with SuperScript VILO (Invitrogen). *Gli1* expression levels were detected with Taqman assays (Invitrogen) and was normalized to Cyclophilin (Primers: GGCCGATGACGAGCCC, TGTCTTTGGAACTTTGTCTGCAA, Probe: TGGGCCGCGTCTCCTTCGA), using StepOne Real-Time PCR System (Thermo Fisher).

### Statistics

p-Values were determined using unpaired, two-tailed student’s *t* test.

## Additional Information

**How to cite this article**: Hibsher, D. *et al*. Pancreatic Mesenchyme Regulates Islet Cellular Composition in a Patched/Hedgehog-Dependent Manner. *Sci. Rep.* **6**, 38008; doi: 10.1038/srep38008 (2016).

**Publisher's note:** Springer Nature remains neutral with regard to jurisdictional claims in published maps and institutional affiliations.

## Figures and Tables

**Table 1:** Estimated β- and α- cells area at e14.5.

	Ins^+^ cell area (μm^2^)	Gluc^+^ cell area (μm^2^)	Ins^+^ cell area/Gluc^+^ cell area
Non transgenic	52 ± 14	46 ± 12	1.13^(*)^
*Nkx3.2*-Cre;*Ptch*^f/f^	48 ± 14	42 ± 11	1.15^(*)^

Cell area (Mean ± SD) was measured in non-transgenic and *Nkx3.2*-Cre;*Ptch*^f/f^ littermate e14.5 embryos. n = 50 cells. (*) p value: p < 0.05, between β-cell and α-cell area within mouse group, determined using Student’s *t*-test. No significance differences were detected between cells area in non-transgenic and transgenic embryos. Ins = insulin; Gluc = glucagon.
